# [^18^F]FDG PET/CT imaging in hyperthyroid myopathy presenting initially as myasthenia: a case report

**DOI:** 10.3389/fmed.2026.1787323

**Published:** 2026-02-11

**Authors:** Na Dang, Yabo Zhao, Jiehuan Wang, Hao Yu, Qingxu Liu, Guanjie Cao, Yueqin Chen, Min Du

**Affiliations:** 1 Department of Medical Imaging, Affiliated Hospital of Jining Medical University, Jining, China; 2 Department of Diagnostic Ultrasound, Affiliated Hospital of Jining Medical University, Jining, China

**Keywords:** case, glucose metabolism, hyperthyroidism, myopathy, PET/CT

## Abstract

**Background:**

Muscle weakness can be a clinical manifestation of a wide range of diseases, often with an obscure etiology and a high risk of misdiagnosis. [^18^F]FDG PET/CT has been widely studied in tumors and inflammatory myopathies; however, reports on [^18^F]FDG PET/CT imaging of hyperthyroid myopathy are exceedingly rare, particularly in patients with a history of malignancy, in whom the diagnostic process is especially challenging.

**Case:**

A 51-year-old woman presented with bilateral lower-limb weakness of more than 1 year’s duration without an obvious precipitating factor. She had undergone surgery and chemotherapy for endometrial carcinoma 2 years earlier. Whole-body [^18^F]FDG PET/CT demonstrated diffusely increased [^18^F]FDG uptake in skeletal muscles throughout the body, with a maximum standardized uptake value (SUVmax) of 5.7. Both thyroid lobes were enlarged, with decreased density and diffusely increased [^18^F]FDG uptake (SUVmax 3.1). Post-treatment changes of endometrial carcinoma were noted, with no evidence of metabolically active tumor recurrence or metastasis. Laboratory tests revealed elevated thyroid hormone levels and a thyroid-stimulating hormone level <0.01 mIU/L. Thyroid ultrasonography showed diffuse enlargement with heterogeneous echotexture and increased vascularity. Electromyography indicated peripheral nerve damage with suspected myogenic involvement. The clinical diagnosis was hyperthyroid myopathy. The patient recovered after treatment with methimazole, leucogen tablets, and propranolol hydrochloride.

**Conclusion:**

In patients presenting with muscle weakness, when whole-body [^18^F]FDG PET/CT shows diffuse hypermetabolism of skeletal muscles mimicking inflammatory myopathy, and concomitant abnormal thyroid metabolism is detected, hyperthyroid myopathy should be considered.

## Introduction

Thyroid disease is one of the etiologies of myopathy. When muscle dysfunction occurs in the presence of overt thyroid disease, the diagnosis is usually straightforward; however, in a small proportion of patients, myopathy may be the initial manifestation of an underlying thyroid disorder (1). The clinical presentation of hyperthyroid myopathy can resemble that of other inflammatory myopathies and neurological diseases, yet the prognosis may be unfavorable. Herein, we focus on the diagnostic process and [^18^F]FDG PET/CT imaging features of a patient with hyperthyroid myopathy who also had a history of malignancy.

## Case report

A 51-year-old woman was admitted on April 21, 2023, with a chief complaint of bilateral lower-limb weakness lasting more than 1 year. One year prior to admission, she developed progressive bilateral lower-limb weakness without an apparent cause, accompanied by difficulty standing, which gradually worsened and impaired ambulation. She denied muscle or joint pain, choking when drinking, or dysphagia. Her medical history was notable for hysterectomy with bilateral oophorectomy for endometrial carcinoma on March 30, 2021, followed by five cycles of postoperative chemotherapy, completed on November 11, 2021.

**Figure 1:**
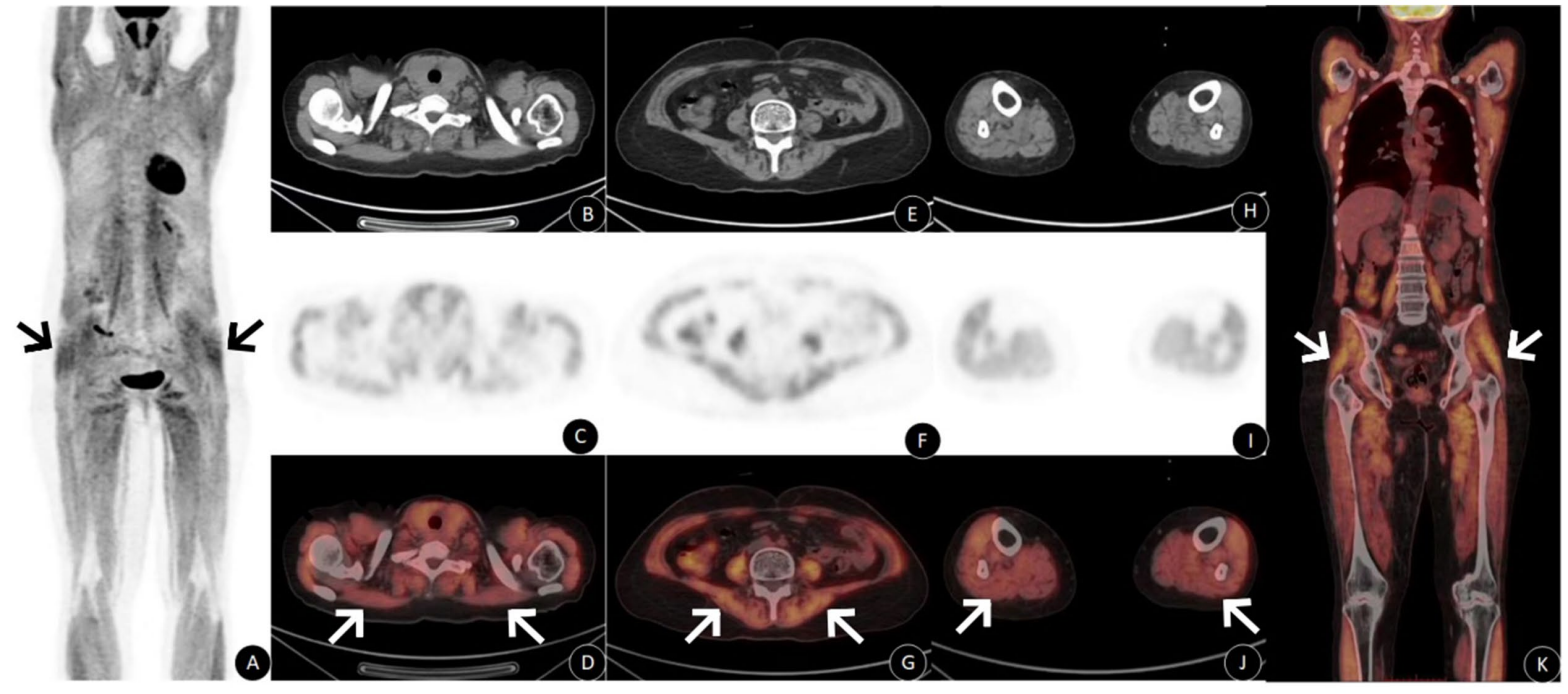
Panel **(A)** shows diffuse increased [^18^F]FDG metabolism in whole body muscles; panel **(B–J)** shows increased muscle metabolism in shoulder, lower back, and lower limbs in PET and PET/CT fusion images, but no obvious abnormality was found in CT images. Panel **(K)** shows the coronal fusion map, and the diffuse metabolism of the whole body muscles is increased.

Physical examination revealed normal muscle strength in both upper limbs (grade 5), decreased muscle tone in both lower limbs, and muscle strength of grade 2 in the lower limbs, without edema. On April 16, 2023, carbohydrate antigen 19–9 was 40.38 U/mL (reference range: 0–37 U/mL). On April 19, 2023, [^18^F]FDGPET/CT whole-body imaging (Figure 1) showed diffusely increased [^18^F]FDG uptake in skeletal muscles throughout the body (SUVmax 5.7). Both thyroid lobes were enlarged, with decreased density and diffusely increased [^18^F]FDG uptake (SUVmax 3.1). Post-treatment changes of endometrial carcinoma were observed, with no evidence of metabolically active tumor recurrence or metastasis.(The patient not have diabetes and should avoid sports, massage and other activities within 48 hoursbefore the examination, have a calm rest, avoid the use of drugs that affect muscle activity, fasting for 4–6 h before the examination, fasting bloodglucose was measured at 5.6 mmol/L).

On April 22, 2023, thyroid function tests showed free triiodothyronine 49.00 pmol/L (reference range: 3.1–6.8 pmol/L), free thyroxine >100.00 pmol/L (reference range: 12–22 pmol/L), and thyroid-stimulating hormone <0.01 mIU/L (reference range: 0.27–4.2 mIU/L). Serum potassium was 2.81 mmol/L (reference range: 3.5–5.3 mmol/L). The myositis panel, high-sensitivity cardiac troponin I, serum myoglobin, immunoglobulins, rheumatoid factor, vasculitis markers, and antinuclear antibodies were all unremarkable. On April 23, 2023, electromyography suggested peripheral nerve damage with suspected myogenic involvement. Thyroid ultrasonography demonstrated diffuse enlargement with heterogeneous echotexture and increased blood flow. On April 24, 2023, thyroid-stimulating hormone receptor antibody was 12.60 IU/L (reference range: 0–1.75 IU/L), anti-thyroglobulin antibody was 675.000 IU/mL (reference range: 0–115 IU/mL), and anti-thyroid peroxidase antibody was >600.000 IU/mL (reference range: 0–34 IU/mL), Creatine kinase: 25 U/L (reference range: 40–200 U/L). ^99m^T_C_O_4_^−^ thyroid scintigraphy showed diffuse thyroid enlargement with globally increased tracer uptake (uptake rate 23.9%, reference range: 0.88–3.75%).

After multidisciplinary consultation, the patient was diagnosed with hyperthyroid myopathy and hyperthyroidism. Treatment consisted of methimazole 10 mg three times daily, leucogen tablets 20 mg three times daily, propranolol hydrochloride 10 mg three times daily, and potassium chloride sustained-release tablets 0.5 g three times daily. The patient’s condition improved markedly, and she was discharged. During follow-up, on June 5, 2023, outpatient examination showed grade 5 muscle strength in all extremities, a normal electromyogram, and complete resolution of symptoms.

## Discussion

Hyperthyroid myopathy is one of the common complications of hyperthyroidism and may present with muscle weakness and muscle atrophy. In some cases, it serves as the initial manifestation and reason for medical consultation, posing diagnostic challenges for clinicians. Common diagnostic criteria for hyperthyroid myopathy include: (1) clinically confirmed hyperthyroidism; (2) slowly progressive muscle weakness with or without muscle atrophy; (3) electromyography and/or muscle biopsy indicating myogenic damage; (4) exclusion of other causes of neuromuscular disease (2).

[^18^F]FDG PET/CT integrates anatomical and functional imaging, providing information on metabolism, molecular activity, and receptor distribution in addition to CT-based morphological changes. Importantly, it enables noninvasive visualization of metabolic alterations before morphological changes become apparent, thereby significantly improving diagnostic accuracy (3, 4). Previous studies have suggested that [^18^F]FDG PET/CT is the most sensitive imaging modality for diagnosing myopathies Muscle biopsy can improve the diagnostic certainty of hyperthyroid myopathy, but it is not a necessary routine examination. Moreover, biopsy of hyperthyroid myopathy may only find minor nonspecific changes, which are mainly used when diagnosis is difficult or treatment is ineffective (5–7). Muscle weakness may also occur in malignancy-associated dermatomyositis, inflammatory myopathies, and paraneoplastic neurological syndromes. Although [^18^F]FDG PET/CT has been widely applied in the screening of malignancies and related complications, as well as in the assessment of inflammatory myopathies, reports on the diagnostic features of [^18^F]FDG PET/CT in hyperthyroid myopathy remain scarce.

Myasthenia in hyperthyroidism myopathy is the result of multiple factors, such as energy metabolism disorder, muscle atrophy caused by accelerated protein decomposition, peripheral nerve damage caused by calcium homeostasis and excitation-contraction coupling disorder, and hypokalemia secondary to thyroid hormone-enhanced sodium-potassium pump activity. However, these factors are part of hyperthyroidism myopathy, not simply accompanying symptoms (8).[^18^F]FDG PET/CT can detect increased metabolic activity in muscles and other potentially involved tissues, such as the thyroid, skin, joints, and lungs, facilitating the timely identification of extramuscular lesions, malignant disease, and muscle inflammation, and providing guidance for subsequent clinical management (9). Other endocrine-related myopathy, such as steroid-induced myopathy, Cushing ‘s syndrome myopathy, hypothyroidism myopathy, primary aldosteronism-related myopathy, showed normal or decreased muscle [^18^F]FDG uptake. Only hyperthyroidism myopathy showed diffuse and symmetrical muscle FDG uptake increased significantly (10, 11). Therefore, the specificity of [^18^F]FDG PET / CT in the diagnosis of hyperthyroid myopathy is very high. Excessive thyroid hormones in patients with hyperthyroid myopathy directly increase the basal metabolic rate and energy consumption demand of the whole body, and the high insulin level caused by hyperthyroidism promotes the increase of GLUT4 transporters on the skeletal muscle cell membrane, thereby increasing the uptake of [^18^F]FDG by the muscle (12). Dffuse FDG uptake in muscle has also been reported in hyperthyroidism without myopathy,compared with this case, the degree of FDG uptake is lower, and the mechanism may be related to the high metabolic state caused by hyperthyroidism (13). In this case, muscle weakness was the initial symptom, and no symptoms of hyperthyroidism occurred. and the patient was evaluated in neurology, rheumatology, immunology, and oncology departments for nearly 1 year without a definitive diagnosis. During this period, abdominal CT and lumbar spine MRI failed to reveal muscle-related abnormalities. Whole-body [^18^F]FDG PET/CT demonstrated diffuse skeletal muscle hypermetabolism and concomitant thyroid abnormalities, while excluding neoplastic myopathy, thereby redirecting clinical attention toward an endocrine etiology.

The cornerstone of treatment for hyperthyroid myopathy is effective control of hyperthyroidism. Once thyroid function is normalized, muscle weakness and atrophy are generally reversible (14). In the present case, the patient achieved complete symptom resolution following antithyroid therapy, with normal muscle strength and electromyographic findings on follow-up. Clinically, some patients with hyperthyroid myopathy present initially with muscle weakness and atrophy, which can be difficult to distinguish from rheumatologic or neurological disorders, leading to misdiagnosis and prolonged disease courses (9).

## Conclusion

This case highlights that when patients present with muscle weakness as the initial symptom in the absence of typical manifestations of thyroid dysfunction, clinicians should remain vigilant for the possibility of hyperthyroid myopathy. Early whole-body [^18^F]FDG PET/CT may assist in excluding or diagnosing malignancy-associated dermatomyositis, inflammatory myopathies, and paraneoplastic neurological syndromes, thereby reducing the risk of misdiagnosis.

## Data Availability

The original contributions presented in the study are included in the article/supplementary material, further inquiries can be directed to the corresponding authors.
